# Dynamic instability of the acromioclavicular joint: A new classification for acute AC joint separation

**DOI:** 10.1007/s11678-018-0469-x

**Published:** 2018-06-28

**Authors:** Natascha Kraus, Carmen Hann, Christian Gerhardt, Markus Scheibel

**Affiliations:** 1 Clinic for Orthopaedics and Orthopaedic Surgery, Center of Orthopaedics, Trauma Surgery and Rehabilitation Medicine, University Medicine Greifswald, Ferdinand-Sauberbruch-Straße, 17475 Greifswald, Germany; 2 Center for Musculoskeletal Surgery, Campus Virchow, Charité-Universitaetsmedizin, Berlin, Germany; 3 Clinic for trauma and hand surgery, St. Vincentius Klinik, Karlsruhe, Germany

**Keywords:** Shoulder, Joint instability, Acromioclavicular joint instability score, Acute dislocation, Rockwood classification, Schulter, Gelenkinstabilität, Akromioklavikulargelenkinstabilitätsscore, Akute Luxation, Rockwood-Klassifikation

## Abstract

**Background:**

Acute acromioclavicular (AC) dislocation is classified according to Rockwood (RW). Although of clinical relevance, dynamic horizontal translation (DHT) is not listed in this classification or in frequently used clinical evaluation tools. The aim of this study was (a) to evaluate vertical and horizontal AC joint instabilities and assess their combined occurrence and clinical appearance in a consecutive group of patients, as well as (b) to develop a new classification of acute AC joint dislocation.

**Method:**

A consecutive group of 61 patients (seven female, 54 male) with a mean age of 34.5 years (18.9–60.1) were included in the study. All patients underwent posttraumatic clinical—Taft Score (TF), Acromioclavicular Joint Instability Score (ACJI), Constant Score (CS), Subjective Shoulder Value (SSV)—and radiological (bilateral anteroposterior stress and bilateral Alexander views) evaluation.

**Results:**

According to the RW classification, the following AC dislocations were present: eight (13.1%) type I, nine (14.8%) type II, 22 (36.1%) type III, and 22 (36.1%) type V. Based on the clinical and radiographic results, a new classification is proposed: Type I instabilities show only a partial vertical displacement (≤30% coracoclavicular distance [CCD]) and type II a complete vertical displacement (>30% CCD). Both type I and II are further graded into none or partial (A) and complete DHT (B) as seen on bilateral Alexander views.

**Conclusion:**

DHT can be found in low-grade instabilities and lead to inferior clinical results in the posttraumatic situation.

## Zusammenfassung

**Hintergrund:**

Akute Akromioklavikulargelenk(ACG-)Sprengungen werden heute mehrheitlich nach Rockwood (RW) eingeteilt. Trotz klinischer Relevanz wird die dynamische horizontale Translation (DHT) weder in dieser Klassifikation noch in vorrangig verwendeten klinischen Evaluationswerkzeugen abgebildet. Das Ziel dieser Studie war es, die vertikalen und horizontalen ACG-Instabilitäten, ihr kombiniertes Auftreten und klinisches Erscheinungsbild an einer konsekutiven Gruppe von Patienten zu untersuchen sowie eine neue Klassifikation akuter ACG-Instabilitäten zu entwickeln.

**Methode:**

Insgesamt wurden 61 Patienten (7 w./54 m.) mit einem mittleren Alter von 34,5 (18,9–60,1) Jahren in diese Studie eingeschlossen. Die klinische und radiologische Evaluation der Patienten umfasste die posttraumatische Erhebung des Taft Score (TF), Acromioclavicular Joint Instability Score (ACJI), Constant Score (CS) und Subjective Shoulder Value (SSV) sowie Panoramaaufnahmen unter Belastung und bilaterale Alexander-Aufnahmen.

**Ergebnisse:**

Gemäß RW-Klassifikation lagen für die ACG-Sprengungen folgende Schweregrade vor: 8‑mal (13,1 %) Typ I, 9‑mal (14,8 %) Typ II, 22-mal (36,1 %) Typ III; 22-mal (36,1 %) Typ V. Basierend auf den klinischen und radiologischen Ergebnissen wurde eine neue Klassifikation erarbeitet: Typ-I-Instabilitäten zeigen eine partielle vertikale (≤30 % CCA, korakoklavikulärer Abstand) und Typ-II-Instabilitäten eine komplette vertikale Translation (>30 % CCA). Beide Typen werden unterteilt in keine oder eine partielle DHT (A) und eine komplette DHT (B), basierend auf bilateralen Alexander-Aufnahmen.

**Schlussfolgerung:**

Eine DHT kann auch bei niedriggradigen ACG-Instabilitäten auftreten und Ursache schlechterer klinischer Resultate in der posttraumatischen Situation sein.

Acute acromioclavicular (AC) joint dislocation is classified according to Rockwood. Although clinically relevant, dynamic horizontal translation is not listed in this classification or in other frequently used clinical evaluation tools. In the present study we describe a new classification of acute AC joint dislocation.

Acute AC joint dislocation is currently classified according to Rockwood into six types based mainly on the coracoclavicular distance (CCD) measured on anteroposterior bilateral stress views and on axillary views to test for static horizontal displacement (Rockwood IV) [12]. Hence, vertical instability has been the major factor in choosing treatment options [6, 7, 13, 14]. A dynamic horizontal translation was thus far not evaluated. However, dynamic horizontal translation has recently been shown to lead to inferior clinical results in patients suffering from a high-grade acromioclavicular joint instability treated with arthroscopically assisted stabilization [10, 14]. A subdivision of Rockwood type III dislocations into type a and b has been published. According to the authors, a type IIIa dislocation represents a horizontally stable situation and a type IIIb an unstable situation [3].

Dynamic horizontal translation is neither displayed in Rockwood’s classification nor in frequently used clinical evaluation tools such as the Taft Score [16]. A new AC joint scoring system, the Acromioclavicular Joint Instability Score (ACJI), includes vertical as well as dynamic horizontal displacement [14]. Since vertical as well as horizontal translation seems to play a role in the clinical appearance, the aim of this study was to evaluate these displacements in a consecutive group of patients and correlate their combined occurrence with the clinical situation leading to a new classification of acute AC joint dislocation.

## Methods

### Patient population

The local ethics committee approved of this study (EA 1/298/12). All patients gave their written informed consent.

From March 2011 to November 2012, all patients who presented to our emergency department or outpatient clinic with an acute AC dislocation (<3 weeks after injury) were included in this study. Patients with an ipsi- or contralateral fracture of the shoulder girdle, a bilateral AC joint injury, or a history of prior shoulder trauma were excluded from this study.

### Radiological evaluation

**Fig. 1:**
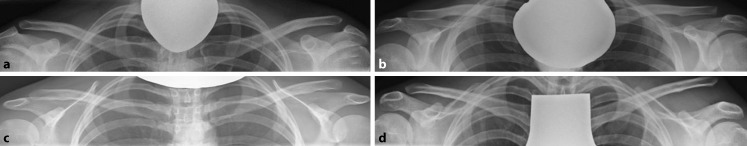
Anteroposterior stress views with 10-kg axial load in order to grade according to Rockwood into type I (**a**), II (**b**), III (**c**), and V (**d**)

**Fig. 2:**
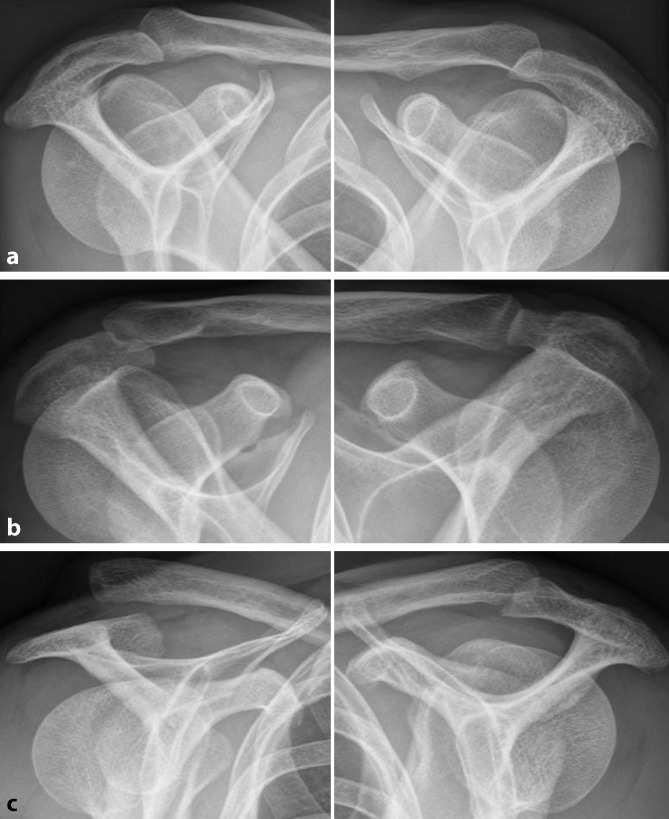
Bilateral Alexander views of affected (left side) and contralateral side (right side) in order to grade into no (**a**), partial (**b**), and complete horizontal translation (**c**)

Radiological evaluation consisted of bilateral anteroposterior stress views with 10 kg of axial load in order to grade injuries according to the Rockwood classification (Fig. 1a–d; [12]). In addition, bilateral Alexander views were obtained to assess the degree of dynamic horizontal translation [1]: A lateral scapular view was obtained with the ipsilateral hand of the patient on the contralateral shoulder (cross-body position). This was classified into no, partial, and complete dynamic horizontal translation (Fig. 2a–c). The lack of horizontal translation is characterized by a clavicle that is in line with the acromion (Fig. 2a). A crossing-over of both bones may be found. However, in comparison with the healthy side, there is no difference regarding posterosuperior translation of the clavicle in relation to the scapula. A partial horizontal translation is present if posterosuperior translation of less than one clavicle width is seen (Fig. 2b). A difference between the two sides is present. However, if both sides show a partial displacement on Alexander views without prior trauma, history of AC joint pathologies, or surgery of the contralateral side, the finding is deemed to be one without horizontal translation. A completely displaced situation is diagnosed if posterior translation of one clavicle width or more is present (Fig. 2c).

### Clinical evaluation

Clinical evaluation consisted of a complete physical examination of the shoulder as well as four shoulder scores: the Taft Score (TF), the ACJI, the Constant Score (CS), and the Subjective Shoulder Value (SSV; [3, 4, 14, 16]).

#### Taft Score

The TF was described by Taft et al. to grade results after conservative and surgical treatment of AC joint dislocations [16]. The maximum score is 12 points. The subcategories are “subjective” (=pain; 4 points), “objective” (=range of motion and strength; 4 points) and “radiologic” (4 points). Furthermore, one point can be subtracted for each of the following: tenderness to palpation of the AC-joint, bad cosmetic results, or crepitation.

#### Acromioclavicular Joint Instability Score

The ACJI was described by Scheibel et al. [14]. In total, 100 points can be achieved in five categories: pain (20 points), activities of daily living (10 points), cosmesis (10 points), function (25 points), and radiological assessment (35 points). Pain is further subdivided into overall shoulder pain (10 points), graded into no pain (10 points), pain during activities (5 points) and at rest (0 points), and tenderness to palpation (10 points), which is divided into present (0 points) or absent (10 points). Regarding the subitem “activities of daily living” (10–5–0 points) no, moderate, and severe impairment can be distinguished. For “cosmesis,” asymmetry of both clavicles is graded by the patient and examiner alike (10–5–0 points). The item “function” evaluates the range of motion (flexion, abduction, external rotation, internal rotation) of the shoulder (10 points) and abduction strength in 90° abduction in the scapular plane on both sides (15 points) using an isometric dynamometer (Isobex TM Dynamometer, MDS AG, Switzerland). The item “radiological assessment” evaluates posttraumatic AC joint arthritis (5–0 points), vertical stability according to Rockwood’s classification (10–8–4–0 points), and horizontal stability (20–10–0 points).

#### Constant Score

The CS is a general outcome measurement tool of shoulder function [3]. It is subdivided into the items “pain” (15 points), “activities of daily living” (20 points), “range of motion” (40 points), and “strength” (25 points). The maximum score is 100 points.

#### Subjective Shoulder Value

The SSV was described as a subjective evaluation score and is defined as the patient’s subjective assessment of the value of her/his affected shoulder given as a percentage of a completely healthy shoulder (=100%; [4]).

### Statistical analysis

Statistical analysis was performed using SPSS version 16.0 (SPSS Inc., Chicago, IL, USA). The Kolmogorov–Smirnov test was used on all data to test for normal distribution. Metric data were compared using the Student *t* test. The results of the CS, TF, SSV, and ACJI were correlated using Pearson’s correlation coefficient and compared employing the Mann–Whitney *U *test. Descriptive results are demonstrated as the mean (range). The level of significance was defined as *p* = 0.05.

Intra- and interobserver reliability was measured.

## Results

### Patient population

In total, 61 patients (seven females/54 males) with a mean age of 34.5 years (18.9–60.1) were included in this study. Patients were seen on average 6.1 days (0–15) after trauma. In 46 patients the dominant side was affected. According to the Rockwood classification, there were eight (13.1%) type I (one female/seven males; mean age 32.0 years [19.4–56.2]), nine (14.8%) type II (nine males; 34.3 years [20.9–60.1]), 22 (36.1%) type III (four females/18 males; 29.8 years [19.5–47.7]), and 22 (36.1%) type V injuries (two females/20 males; 37.7 years [18.9–55.4]; Table 1). AC joint dislocation type IV as originally described by Rockwood being a static posteriorly displaced clavicle was not found [12]. According to the ISAKOS modification, there were 14 type IIIa and eight type IIIb injuries.

**Table 1:** Population characteristics

Injury type	*N*	F	M	Mean age, years (range)	d	nd
I	8	1	7	32.0 (19.4–56.2)	7	1
II	9	0	9	34.3 (20.9–60.1)	1	8
III	22	4	18	29.8 (19.5–47.7)	13	9
V	22	2	20	37.7 (18.9–55.4)	15	7
Total	61	7	54	34.1 (18.9–60.1)	36	25

*N* number, *F* female, *M* male, *d* dominant, *nd* nondominant

### Radiological results

All patients were able to partake in the aforementioned radiological evaluation. No patient was unable to complete the radiologic evaluation because of pain or any other reason. The CC distance of the affected side averaged 11.7 mm (8–16) in Rockwood type I, 13.3 mm (10.6–16) in Rockwood type II, 15.1 mm (9–24) in Rockwood type III, and 22.4 mm (13–29.8) in Rockwood type V injuries.

Signs of posterior dynamic translation were noted in 43 patients (Table 2). In total, 18 patients had no (I: *n* = 5; II: *n* = 6; III: *n* = 7; V: *n* = 0), 13 had partial (I: *n* = 2; II: *n* = 3; III: *n* = 7; V: *n* = 1), and 30 had complete (I: *n* = 1; II: *n* = 0; III: *n* = 8; V: *n* = 21) dynamic posterior translation on bilateral Alexander views in comparison with the contralateral side. Signs of dynamic horizontal translation were noted in grade I and II injuries and were not strictly associated with high-grade dislocation.

**Table 2:** Dynamic horizontal translation

Injury type	None	Partial	Complete
I	5	2	1
II	6	3	0
III	7	7	8
V	0	1	21
Total	18	13	30

### Clinical results

Patients scored on average 5.4 points (2–10) in the TF, 33.3 points (5–80) in the ACJI, 52.4 points (13–91) points in the CS, and 45.3% (0–92) in the SSV (Table 3).

**Table 3:** Score results

Injury type	CS, points (range)	SSV, % (range)	TF, points (range)	ACJI, points (range)
I	68.2 (26–88)^a,b^	71.0 (50–85)^c,d^	8.3 (7–10)^e,f^	66.7 (55–80)*
II	61.9 (32–91)	51.7 (20–92)	7.1 (6–9)^g,h^	51.2 (28–78)*
III	47.4 (13–78)^a^	40 (0–90)^c^	5.2 (2–9)^e,g^	33.0 (9–64)*
V	48.6 (23–66)^b^	40.6 (20–70)^d^	4.1 (2–6)^f,h^	16.4 (5–35)*
Total	52.4 (13–91)	45.3 (0–92)	5.4 (2–10)	33.3 (5–80)

*CS* Constant Score, *SSV* Subjective Shoulder Value, *TF* Taft Score, *ACJI* Acromioclavicular Joint Instability Score

^a–h^Statistically significant difference between the pairs, *p* < 0.05; *statistically significant difference between all types of injury, *p* < 0.05

Patients with a Rockwood type I injury achieved 8.3 points (7–10) in the TF (Table 4), 66.7 points (55–80) in the ACJI, 68.2 points (26–88) in the CS (Table 5), and 71.0% (50–85) in the SSV. Rockwood type II injuries reached 7.1 points (6–9) in the TF, 51.2 points (28–78) in the ACJI, 61.9 points (32–91) in the CS, and 51.7% (20–92) in the SSV. Patients suffering from a type III injury scored 5.2 points (2–9) in the TF, 33.0 points (9–64) in the ACJI, 47.4 points (13–78) in the CS, and 40% (0–90) in the SSV. The group of patients with a Rockwood type V injury achieved 4.1 points (2–6) in the TF, 16.4 points (5–35) in the ACJI, 48.6 points (23–66) in the CS, and 40.6% (20–70) in the SSV. The ACJI was the only score that was able to distinguish significantly between all the types of AC joint dislocation (*p* < 0.05; Table 6).

**Table 4:** Taft Score results

Injury type	Subjective, points (range)	Objective, points (range)	Radiologic, points (range)	Total, points (range)
I	2.4 (2–3)^a^	2 (1–3)^c^	3.6 (3–4)*	68.2 (26–88)^e,f^
II	2.3 (2–3)^b^	1.7 (1–3)^d^	3.1 (3–4)*	61.9 (32–91)^g,h^
III	2.1 (1–3)	0.8 (−1–4)	2.6 (2–4)*	47.4 (13–78)^e,g^
V	1.9 (1–3)^a,b^	0.2 (−1–3)^c,d^	2 (2)*	48.6 (23–66)^f,h^
Total	2.1 (1–3)	0.9 (−1–4)	2.6 (2–4)	52.4 (13–91)

^a–h^Statistically significant difference between the pairs, *statistically significant difference between all types of injury, *p* < 0.05

**Table 5:** Constant Score results

Injury type	Pain, points (range)	ADL, points (range)	ROM, points (range)	Strength, points (range)	Total, points (range)
I	11.8 (10–14)	10.8 (8–13)	34.3 (12–40)^a^	11.4 (0–21)^b,c^	68.2 (26–88)^d,e^
II	10.8 (8–13)	8.9 (6–15)	32.9 (16–40)	9.1 (0–25)	61.9 (32–91)
III	10.6 (6–15)	6.8 (2–14)	26.5 (4–40)^a^	4.8 (0–21)^b^	47.4 (13–78)^d^
V	9.2 (4–15)	7.3 (4–20)	29.2 (12–40)	4.7 (0–11)^c^	48.6 (23–66)^e^
Total	10.3 (6–15)	7.8 (2–20)	29.4 (4–40)	6.2 (0–25)	52.4 (13–91)

*ADL* activities of daily living, *ROM* range of motion

^a–e^ Statistically significant difference between the pairs

**Table 6:** ACJI score results

Injury type	Pain, points (range)	ADL, points (range)	Cosmesis, points (range)	Function, points (range)	Radiology, points (range)	Total points (range)
I	6.3 (5–15)	4.3 (0–5)	8.1 (0–10)^b,c^	14.4^g,h^ (0–25)	30^i,j^ (15–35)	66.7 (55–80)*
II	4.4 (0–10)	5^a^ (5)	5 (0–10)^d,e^	8.9 (0–25)	29.7^k,l^ (23–33)	51.2 (28–78)*
III	4.8 (0–15)	3.9 (0–10)	1.1 (0–10)^b,d,f^	5.5^g^ (0–20)	18.5^i,k,m^ (9–29)	33.0 (9–64)*
V	4.3 (0–15)	2.7^a^ (0–5)	0 (0)^c,e,f^	3.6^h^ (0–15)	5.2^j,l,m^ (0–15)	16.4 (5–35)*
Total	4.8 (0–15)	3.7 (0–10)	2.2 (0–10)	6.5 (0–25)	16.9 (0–35)	33.3 (5–80)

*ADL* activities of daily living, *ACJI* Acromioclavicular Joint Instability Score

^a–m^Statistically significant difference between the pairs, *p* < 0.05; *statistically significant difference between all types of injury, *p* < 0.05

### Correlation between clinical and radiological results

Regarding radiographic signs of vertical and dynamic horizontal translation and their correlation with clinical results, patients with high-grade AC joint instability (Rockwood type III and V) scored significantly worse in all scores in comparison with patients with low-grade instabilities (type I and II). Interestingly, however, there was no significant difference in overall score results between Rockwood type III and V. Between type III and V injury, only radiographic and cosmetic subitems of the ACJI were significantly different (*p* < 0.05). There was no statistically significant difference between a type III and type V injury in the CS, SSV, and TF. However, if vertical displacement is graded into a CCD of ≤30% and >30%, a significant difference regarding the clinical appearance can be found. Furthermore, patients with dynamic horizontal displacement scored significantly worse in all scores than did patients who showed no or partial dynamic horizontal translation (*p* < 0.05). Regarding type III injury, those patients who had no signs of horizontal translation scored on average 6.5 points (3–9) in the TF, 42.8 points (29–64) in the ACJI, 50.9 points (13–77) in the CS, and 41.4% (10–70) in the SSV.

Those who showed partial translation reached a mean TF score of 6.4 points (3–9), a mean ACJI score of 41.1 points (24–54), a mean CS score of 61.4 points (37–78), and an SSV of 57.1% (10–90). The eight patients with complete horizontal displacement scored worse than both other groups in all scores—TF: 3.8 points (2–5), ACJI: 20.1 points (9–34), CS: 38.9 points (20–68), SSV: 31.3% (0–60).

Except for the radiologic assessment of the ACJI, there were no statistically significant differences in the score results between horizontally stable and partially displaced injuries (*p* > 0.05). Complete horizontal displacement scored significantly worse in comparison with both other groups in the AC joint specific scores and significantly worse than the partially displaced injury group. Thus, it seems that partial dynamic posterior translation shows a similar clinical situation to isolated one-directional vertical instability.

### New classification system

Based on both the clinical and radiologic results, a new classification of acute injuries of the A‑C‑C (Acromio-coraco-clavicular) complex is proposed: Type I instabilities show only a partial vertical displacement (≤30% CCD) and type II a complete vertical displacement (>30% CCD). Both type I and II are further graded into no or partial dynamic horizontal translation (A) and complete dynamic horizontal translation (B) as seen on bilateral Alexander views (Table 7). Intra- and interobserver reliability showed substantial to high correlation (κ = 0.82/0.80).

**Table 7:** New classification of acute AC joint instability

Type I: Partial vertical displacement (CCD ≤ 30%)	A: None/partial dynamic horizontal translation
	B: Complete horizontal dynamic translation
Type II: Complete vertical displacement (CCD > 30%)	A: None/partial dynamic horizontal translation
	B: Complete dynamic horizontal translation

*CCD* coracoclavicular distance

## Discussion

Acute AC joint dislocation leads to sprain and/or rupture of the AC and/or coracoclavicular ligaments and therefore presents a soft tissue injury. Today, a classification based on the measurement of the distance between two bony structures on anteroposterior radiographs with 10 kg of axial load is used to grade the degree of this ligamentous injury and to decide on treatment options [12]. Based on this, conclusions regarding the degree of ligamentous injury and treatment decisions are made. However, to what extent clinical severity is thoroughly displayed in this classification is yet unknown.

This study presents the clinical and radiological data of a large patient cohort with acute AC joint instability, based on the Rockwood classification. The data showed that bidirectional dynamic instability in the vertical as well as horizontal plane is not associated with high-grade injury only but can also be seen in Rockwood type I and II injuries. Furthermore, dynamic horizontal translation on Alexander views presents a risk factor for inferior clinical results in the acute posttraumatic situation, which was not previously known. Furthermore, it seems that partial dynamic horizontal translation does not lead to significantly worse clinical results, but instead shows a similar situation to isolated one-directional, vertical instability. Both have been shown for persisting dynamic horizontal translation after surgical AC joint repair [8, 14]. However, to date, this was not known in the posttraumatic setting. Further studies are needed to determine whether this presents a risk factor for ongoing problems after AC joint instability.

In high-grade AC injury, no clinical difference in score results was found between type III and type V injury. Differences between type III and V injury were seen in the ACJI only, owing to its radiologic subitem based on Rockwood’s classification. These facts raise doubts regarding the clinical significance of this widely used classification. Therefore, a new classification system of A‑C-C injury is proposed (Table 7).

Type I instabilities show only a partial vertical displacement (≤30% CCD) and type II a complete vertical displacement (>30% CCD). Both type I and II are further graded into no or partial dynamic horizontal translation (A) and complete dynamic horizontal translation (B) as seen on bilateral Alexander views.

In this study, we encountered far more high-grade injuries than type I and II injuries. This contrasts with Rockwood’s observation. His institution recorded 185 (36%) type I, 119 (23%) type II, 204 (39%) type III, four (0.7%) type IV, seven (1.2%) type V, and one (0.1%) type VI injuries of a total of 520 AC joint separations in a 5-year period [12].

The reason for this is yet unknown, since we classified injuries in accordance to Rockwood’s proposed anteroposterior stress views with a 10-kg axial load [15]. Of course, we only have data available of patients who presented either in the emergency department or in the outpatient clinic. Our frequency distribution may therefore be tainted as patients suffering from a type I injury might not present to a hospital. However, in the Rockwood classification there is a lack of information regarding the described extent of ligamentous injury and the display of these injuries on radiographs. When describing AC and coracoclavicular ligament injury in his classification, Rockwood accurately describes and distinguishes differing grades of disruptions. However, radiographic aspects remain vague. A type III injury is seen as a complete dislocation of the clavicle in comparison with the acromion. Similarly, a type V injury is also seen as such, but with a two- or threefold increased CCD. In how far both aspects of this classification fit together remains unclear.

Furthermore, we did not encounter any Rockwood type IV injuries with a static horizontal dislocation of the clavicle during the observation period. Therefore, no prediction regarding clinical appearance and score results can be made. However, as described before, even Rockwood stated that a type IV injury is relatively rare and he found them in only 0.7% of cases [12]. He proposes operative therapy for all type IV injuries regardless of their vertical dislocation, as the static dislocation posteriorly into the trapezius muscle needs to be reduced. Rockwood specifically distinguishes between a static posterior dislocated type IV injury and a dynamic posterior translation as seen on bilateral Alexander views [12].

### Limitations

Of course, this study has some limitations. First, pain in the acute posttraumatic setting has an important influence on other clinical assessment items such as range of motion or strength. We do not know in how far the score results might have been different without the influence of pain.

However, on average, patients were seen 6 days after trauma, by which time most acute pain should have subsided. Furthermore, pain medication might constitute a bias regarding score results for pain in the acute setting. None of the patients received a continuous analgesic treatment. Instead, patients were advised to take pain medication when necessary. Thus, the effect on the scores is hard to validate.

Secondly, we graded injuries according to Rockwood on radiographs. There is conflicting evidence in the literature regarding the reliability of the Rockwood classification [2, 5, 9]. As mentioned before, this was one of the reasons we proposed a new classification that correlates with the clinical presentation of patients in the posttraumatic setting. In how far this classification can be predictive of treatment indications remains to be shown in follow-up studies.

Thirdly, exact radiographic evaluation is limited by standardized patient positioning. The axillary view has been known to be highly examiner-dependent and unreliable, whereas in our experience Alexander views can be obtained in a reproducible fashion as described earlier. This was one of the reasons we chose them as second views in our study and classification rather than axillary stress views. Besides, axillary stress views as described by Tauber et al. require three radiographs of each side [17]. We found that bilateral Alexander views need less radiation exposure and are easier to learn by radiology assistants. Furthermore, recently a very promising quantification method for Alexander views was proposed by Minkus et al. that involves measuring the overlapping length of the acromion and clavicle et al. [11]. We did not apply this method in our study. We felt it might be easier to use a classification with one measurement and one dichotomous variable.

Finally, a limitation of this study is that horizontal translation is more often associated with high-grade AC instability. Therefore, patients displaying a dynamic horizontal translation might score clinically worse because of their high-grade CC injury and not because of horizontal translation only. However, regarding type III, the same effect can be witnessed without a difference in CC distance. Thus, dynamic horizontal translation plays a role in inferior clinical results not only in the postoperative follow-up, but also in the acute, posttraumatic situation.

## Practical conclusion

- Posttraumatic clinical and radiologic findings of acute AC joint instability are heterogeneous and do not always follow Rockwood’s classification.
- This study found posterior dynamic translation to also be present in low-grade instabilities and found inferior clinical results in the posttraumatic situation in patients displaying a dynamic posterior translation.
- Based on these findings, a new classification system is proposed.
